# The prevalence of substance use among Russian, Somali and Kurdish migrants in Finland: a population-based study

**DOI:** 10.1186/s12889-018-5564-9

**Published:** 2018-05-22

**Authors:** Essi Salama, Solja Niemelä, Jaana Suvisaari, Tiina Laatikainen, Päivikki Koponen, Anu E. Castaneda

**Affiliations:** 1 Doctoral Programme in Clinical Research, Faculty of Medicine, University of Turku, Kiinamyllynkatu 10, FI-20520 Turku, Finland; 2 Department of Child Psychiatry, Turku University Hospital and University of Turku, Building 10, Kiinamyllynkatu 4-8, FI-20521 Turku, Finland; 3 Research Unit of Clinical Neuroscience, University of Oulu, P.O.Box 8000, FI-90014 University of Oulu, Oulu, Finland; 4 Department of Psychiatry, University of Turku, FI-20014 Turku, Finland; 5 National Institute for Health and Welfare (THL), P.O. Box 30, FI-00271 Helsinki, Finland; 6 Institute of Public Health and Clinical Nutrition, University of Eastern Finland, P.O. Box 1627, FI-70211 Kuopio, Finland; 7 Joint municipal authority for North Karelia social and health services, Tikkamäentie 16, 80210 Joensuu, Finland; 8 Faculty of Medicine, Department of Psychology and Logopedics, University of Helsinki, Haartmaninkatu 8, P.O. Box 63, FI-00014 Helsinki, Finland

**Keywords:** Migrant, Migration, Substance use, Alcohol, Cigarette smoking, Cannabis, Population-based study, Nordic countries

## Abstract

**Background:**

Substance use is a well-known public health problem, but population-based research on migrants’ substance use in Europe is limited. Factors related to the cultural background and current life situation might influence substance use among migrants. Here, the prevalence of substance use in Russian, Somali and Kurdish migrants in Finland is reported in comparison with the general population, and the associations between substance use and socio-economic and migration-related background factors among migrants are analysed.

**Methods:**

Cross-sectional data from the Finnish Migrant Health and Wellbeing Study (Maamu) and comparison group data of the general Finnish population (*n* = 1165) from the Health 2011 Survey were used. The survey participants were of Russian (*n* = 702), Somali (*n* = 512), and Kurdish (*n* = 632) origin. Substance use included self-reported alcohol use within previous 12 months (AUDIT-C questionnaire), current and lifetime daily smoking and lifetime use of cannabis and intravenous drugs.

**Results:**

Binge drinking was less prevalent among all migrant groups than in the general Finnish population (Russian men 65%, *p* < 0.01; Russian women 30%, *p* < 0.01, Somali men 2%, *p* < 0.01, Kurdish men 27%, *p* < 0.01, Kurdish women 6%, *p* < 0.01, general population men 87% and women 72%). Current daily smoking was more prevalent among Russian (28%, *p* = 0.04) and Kurdish (29%, *p* < 0.01) migrant men compared with the reference group (20%). Younger age and employment were associated with binge drinking among migrants. Socio-economic disadvantage increased the odds for daily smoking in Russian, Somali and Kurdish migrant men. Several migration-related factors, such as age at migration and language proficiency, were associated with substance use.

**Conclusions:**

Binge drinking is less common among migrants than in the Finnish general population. However, current daily smoking was more prevalent among Russian and Kurdish migrant men compared with the general population. Younger age, level of education, employment, duration of residence in Finland and language proficiency were associated with binge drinking and daily smoking with varying patterns of association depending on the migrant group and gender. These findings draw attention to the variation in substance use habits among migrant populations.

## Background

Substance use is a well-known risk factor for disease, e.g. cardiovascular diseases and cancers, and premature mortality worldwide [1–4]. Recent evidence from the Global Burden of Diseases, Injuries, and Risk Factors Study 2016 showed that smoking was globally the leading risk factor in terms of attributable disability-adjusted life years in men, while alcohol use ranked fourth [5]. For women smoking and alcohol were among the 20 leading risk factors for disease and premature mortality [5]. Similarly, a range of adversities has been associated with frequent cannabis use, particularly with an early onset in adolescence [6, 7]. Despite its significant burden on individuals and society, research on substance use has remained a low priority in particular concerning migrant populations [8]. Little information is available on the substance use of adult migrant populations compared with the general population in the European context, especially regarding the association between substance use and socioeconomic and migration-related factors.

Traditional cultural patterns of substance use in migrant populations may evolve due to acculturation and other factors after immigration to a new country [9]. Alternatively, maintaining the substance use habits of the country of origin can become an important part of the individual’s ethnic identity. Migrants are exposed to vulnerabilities, such as stressful life situations, lack of social support and socioeconomic disadvantage, more often than the general population [10]. These vulnerabilities are often associated with substance use, and it has been suggested that especially young migrants of ethnic minorities are more prone to develop risky behaviours due to the acculturative stress and encountering discrimination and prejudices [11].

Population-based research on migrant’s substance use in European context is scarce, although population-based research design allows for taking the diversities between migrant populations into account. In addition, to our knowledge the prevalence of alcohol use and illicit drug use among Kurdish migrants has not been reported before. According to previous literature, alcohol use among migrants in Europe seems to depend on the region of origin, and alcohol consumption and binge drinking among non-European migrants seems to be lower than in the general population [12–17]. The smoking rates of migrant populations in comparison with the general populations in European studies seem to vary according to migrant groups studied, and depend on the region of origin, gender and age of the migrants [12, 15, 16, 18]. The findings of European studies reporting the prevalence of cannabis use among migrant adolescents of non-European origin compared with the general population adolescents are contradictory [12, 13, 19]. Information on substance use among Kurdish migrants is limited. To our knowledge, the prevalence of smoking and excess alcohol consumption among Kurdish migrants has only been reported in a Finnish study examining cardiovascular risk factors among older migrants in Finland [20, 21]. However, different methodologies used in previous research, hinder comparing the results between different studies. Based on previous research, it has been proposed that migration-related phenomena, such as acculturation, the role of native cultural and social context, education, and generational differences, influence the substance use of migrant populations [14, 17, 19, 22].

In contrast to other European countries, migration to Finland has started later and the proportion of migrants in the population is lower. The migrant groups selected to this study represent different migration backgrounds: Russian participants are mainly voluntary migrants having migrated due to personal or work-related reasons, whereas Somali and Kurdish participants are forced migrants having migrated due to humanitarian reasons [23]. Migrants from Russia and other areas of the former Soviet Union form the largest migrant group in Finland [24]. In Russia, alcohol consumption per person is among the highest globally [25] and the prevalence of smoking is also higher than in other European countries, especially among men [1, 26, 27]. Somali migrants are the largest group of refugees and asylum seekers in Finland, and they represent a population where the majority are Muslim by religion. In contrast to Russians, abstaining from alcohol use is common [28], and the prevalence of daily smoking among men is similar to European men, but lower in women [1, 26, 27]. Kurdish is one of the largest foreign-language groups in Finland, and the majority of the recently accepted quota refugees in Finland were from Iraq and Iran when the survey was planned [23]. Specific information about the prevalence rates of alcohol use and smoking in the Kurdistan area or among Kurdish migrants is very limited, but the prevalence of lifetime abstainers from alcohol in Iran and Iraq has been reported to be high [28, 29]. The prevalence of daily smoking is similar or higher in men in Eastern Mediterranean Region compared with European countries but much lower in women [1, 26, 27, 30–32]. However, there are also contradictory findings reporting lower rates of daily smoking in Iran and Iraq compared to the European general estimates [33, 34]. The reported national annual prevalence rates of cannabis use are fairly similar in Finland, Russia, Iran and Somalia [35]. The Finnish alcohol use and smoking habits differ from the general European estimates to some extent: heavy episodic drinking is more common among both genders, while men smoke less compared to the general European estimates [33, 34, 36].

Previous literature and the public health importance of substance use highlight the need for population-based studies reporting substance use among adult migrant populations by ethnicity in the European context.

## Methods

### Aim

In this study, we aim to report the prevalence of alcohol use, cigarette smoking and consumption of illicit drugs among three migrant groups in Finland in comparison with the Finnish general population. In addition, our aim is to study which socio-economic and migration-related background factors are associated with substance use among population groups studied.

### Study design and procedures

The data used were from a comprehensive cross-sectional survey, the Finnish Migrant Health and Wellbeing Study (Maamu) [23, 37], (Castaneda AE, Rask S, Härkänen T, et al.: Enhancing survey participation among migrant populations by development of recruitment strategies and fieldwork protocols. Experiences from the Finnish Migrant Health and Welfare Study (Maamu), submitted), carried out by the Finnish National Institute for Health and Welfare (THL) during 2010–2012, and targeted at migrants of Russian, Somali, and Kurdish origins. Migrant was defined as having been born outside Finland and having residence permit in Finland.

The Maamu survey consisted of a health examination and a structured face-to-face interview on health and wellbeing, conducted by trained field staff in the participants’ native language or in Finnish. The personnel were of Russian, Somali or Kurdish origin, and thus were proficient in both Finnish and the participants’ native language. A short interview including the most essential items of the interview and health examination was generated to reach those who were unable to participate in the interview and health examination.

### Participants

The Maamu survey sample consisted of 3000 migrants aged 18–64 years, with 1000 participants from each of the defined migrant groups. A stratified random sample in six towns (Helsinki, Espoo, Vantaa, Turku, Tampere, Vaasa) was drawn from the National Population Register. Russian origin was defined by the native language being Russian or Finnish and the country of birth being Russia or the Former Soviet Union. Somali origin was defined by the country of birth being Somalia. Kurdish origin was defined by the native language being Kurdish and the country of birth being Iraq or Iran. Persons who had been residents in Finland for less than one year were excluded from the sample. Asylum seekers are not registered in the National Population Register until residence permit is granted, thus persons in asylum seeking process are not included in the sample. A detailed description of the Maamu survey and its data collection methodology has been reported elsewhere [37].

The data of the general Finnish population were obtained from the Health 2011 Survey, also conducted by the National Institute for Health and Welfare [38] and collected at the same time with similar methods. The comparison group consisted of participants from the same municipalities and the same age group as in the Maamu study. The total participation rate of the Health 2011 survey in these municipalities was 64%, and responses to the items on substance use were available for 51% (*n* = 1165) of the sample.

### Ethical approval

Ethical approval was granted to both studies (Maamu and Health 2011) by the Coordinating Ethics Committee of the Hospital District of Helsinki and Uusimaa. Written informed consent was obtained from each participant in the health examination and interview. In the case of participating only in the short interview, oral consent was obtained from those participating face-to-face or by phone, and for those answering by mail, returning the filled short questionnaire was interpreted as a consent among those answering by mail.

### Measurements

#### Alcohol use

As a screening question in the interview and short interview, the participants were asked if they had used alcohol during the previous 12 months (yes vs. no). Alcohol use was measured with the three-item AUDIT-C questionnaire, a short version of the 10-item AUDIT-questionnaire. AUDIT-C has been found to be valid for screening alcohol problems among adults [39, 40], and also of different ethnicities [41]. The frequency of alcohol use was measured by the item “How often do you have a drink containing alcohol?”, which was analysed as three-class variable: “Never” combining the negative response to the previous screening question; “Occasionally” including “Monthly or less” and “2 to 4 times a month”; “Frequently” including “2 to 3 times a week” and “4 or more times a week”. The frequency of alcohol use was included in the short interview. The usual number of alcohol units consumed was analysed as a four-class variable: “No alcohol use”; “1 to 2 units”; “3 to 6 units”; and “7 units or more”. The frequency of binge drinking (consuming six or more drinks on one occasion) was analysed as four-class variable: “No alcohol use”; “No binge drinking”; “Occasional” including “Less than monthly” and “Monthly”; “Frequently” including “Weekly” and “Daily or almost daily”. The usual amount of alcohol units consumed and frequency of binge drinking were not included in the short interview. Binge drinking was selected as the outcome variable for the logistic regression analyses and dichotomised (binge drinking yes vs. no). A total sum score for the AUDIT-C questionnaire with cut-off limits of 4 for women and 6 for men was applied to indicate risky drinking [42]. In the Health 2011 survey the AUDIT-C questionnaire was included in the self-administered questionnaire.

#### Cigarette smoking

The items of cigarette smoking were collected in the interview and short interview. Lifetime regular smoking was measured by a question “Have you ever smoked regularly (every day for at least a year)?” (yes vs. no). Frequency of current smoking (“Do you smoke currently (cigarettes, cigars, pipe)?)”. was included in the short interview and measured as a three-class variable: “No” including also the negative responses to the previous item; “Occasionally”; “Daily”. The quantity of cigarette products (manufactured or self-rolled cigarettes) consumed per day was analysed as a three-class variable: “No smoking” according to the previous responses; “1 to 10”; “11 or more”. The variable measuring the frequency of current smoking was selected as an outcome variable for the logistic regression analyses, and dichotomised (“daily smoking” including answer “yes, daily” vs. “no” including answers “occasionally” and “no”). No data on the general population were available in a fully comparable form for the item of quantity of cigarettes consumed.

#### Illicit drug use

Lifetime use of cannabis (yes vs. no) was asked in the interview, and it was followed by a follow-up item about cannabis use during the previous 12 months (yes vs. no). Lifetime use of intravenous drugs (yes vs. no) was asked in the interview. Lifetime use of cannabis was selected as an outcome variable for the logistic regression analyses. No data on the general population were available for these items.

### Socio-demographic variables

The socio-demographic background variables used were sex, age group (18 to 29; 30 to 45; 46 to 65 for binge drinking and daily smoking, and 18 to 29; 30 to 65 for lifetime cannabis use), marital status (married or cohabitating vs. other), level of basic education (secondary school or less corresponding to the 9 years of compulsory education for Finnish citizens vs. higher), employment (employed; unemployed; economically inactive) and a subjective evaluation of the economic situation (satisfactory vs. unsatisfactory). No general population data were available for the item of economic situation.

The migration-related background variables selected were age at migration to Finland (underage vs. 18 years or more), duration of residence in Finland (5 years or less vs. > 5 years), grounds for the residence permit (refugee or asylum seeker vs. other) and self-reported language proficiency of the local languages (Finnish or Swedish) (good vs. fair or less than fair). Duration of residence, age at migration and language proficiency were considered to describe acculturation.

### Data management and statistical analyses

The statistical analyses were performed with Stata software version 13 IC, and the survey’s sampling design was taken into account in all analyses. The effects of missing data were accounted for using inverse probability weighting. Weights were determined by the main predictive factors of nonresponse: migrant group, sex, age, research municipality and marital status. Finite population correction was applied because of the relatively small population sizes and inclusion of significant proportion of the total population. Adjustment for age was used in all the reported analyses because of the significantly differing age distribution between the studied groups, but all preliminary analyses were also conducted without the age-adjustment [43].

The age-adjusted prevalences for substance use were reported separately for men and women in each migrant group and for the general population. The comparisons were made between men and women in each migrant group, and between each migrant group and the general population by gender. The frequencies were determined using cross-tabulation and the age-adjusted proportions were produced using logistic regression analysis and predictive margins [44].

The associations between the background and substance use variables were analysed using univariate logistic regression analysis adjusting for age. The analyses were conducted separately for each migrant group by sex, each explanatory background variable (age; marital status; level of basic education; employment status; economic situation; age at migration; duration of residence in Finland; language proficiency) and each outcome variable (binge drinking; daily smoking; lifetime use of cannabis). The odds ratios (OR) and 95% confidence intervals (CI) are reported as age-adjusted figures, and a *p* < 0.05 was considered to be statistically significant.

## Results

Altogether, 1846 individuals (62%) took part in at least some part of the survey, with a sufficient response rate to the items on substance use (Russian 69% (*n* = 688); Somali 48% (*n* = 475); Kurdish 61% (*n* = 613)). Because all of the items were not included in the short interview, the total number of participants varies between items in the tables.

The characteristics of the study population are presented in Table 1. The majority of the Somali (99%) and Kurdish participants (75%) reported to be Muslim and nearly half of the Russian participants (49%) reported to be orthodox Christian by religion. Almost a third of the Russian and a fifth of the Kurdish participants reported not belonging to any religion. The majority of Somali (72%) and Kurdish (75%) participants had migrated as refugees or asylum seekers, whilst the majority of Russian migrants (99%) had other grounds for their residence permit including family ties and employment.

**Table 1:** Descriptive statistics of the study population by ethnicity adjusting for age

	Russian			Somali			Kurdish			General population	
	n	%	p*	n	%	p*	n	%	p*	n	%
Gender											
Men	251	37	< 0.01	211	45	0.32	325	56	< 0.01	493	48
Women	437	63		264	55		288	44		672	52
Age											
18 to 29	181	27	< 0.01	197	41	< 0.01	215	37	< 0.01	288	37
30 to 45	252	36		189	42		277	45		351	28
Over 45	255	37		89	18		121	18		526	36
Marital status											
Married or cohabiting	443	60	0.96	313	66	0.07	422	65	0.03	752	62
Basic education											
High school graduate	517	77	< 0.01	108	28	< 0.01	253	42	< 0.01	783	66
Employment situation											
Employed	383	54	< 0.01	104	25	< 0.01	237	40	< 0.01	829	69
Unemployed	137	22		91	20		155	25		48	4
Economically inactive	167	25		266	55		218	36		273	27
Economic situation											
Unsatisfactory	296	45		299	62		396	65	0.00**		
Age at migration											
Underage	150	24		187	40		138	24	0.00**		
Duration of residence in Finland											
> 5 years	550	81		350	81		488	80	0.96**		
Grounds for residence permit											
Refugee or asylum seeker	4	0		229	72		377	75	0.00*		
Language proficiency											
Good	386	65		288	81		273	52	0,00**		

*between the migrant group and general population

**over the migrant groups

### The prevalence of alcohol use

The age-adjusted prevalences of substance use by ethnic group and sex are presented in Table 2. The proportion of abstainers was large among Kurdish men (51%) and women (85%). The frequency of alcohol use was significantly higher among men than women in Kurdish migrants and the general population, but not among Somali or Russian migrants (Table 2). Almost all (men 98%, women 100%) Somalis reported abstaining from alcohol use during the previous year, and thus further analyses could not be performed in the Somali group.

**Table 2:** Age-adjusted prevalences of substance use by ethnicity and gender^1^

	Russian						Somali						Kurdish						General population			
	Men			Women			Men			Women			Men			Women			Men		Women	
	n	%	p*	n	%	p*	n	%	p*	n	%	p*	n	%	p*	n	%	p*	n	%	n	%
Frequency of alcohol use																						
None	32	11	< 0.01	71	16	< 0.01	208	98	< 0.01	263	100	< 0.01	172	51	< 0.01	244	85	< 0.01	39	7	53	8
Occasional	186	73		310	70		3	2		1	0		143	45		44	15		233	53	439	66
Frequent	33	16		56	14		0	0		0	0		10	3		0			221	39	180	25
p**						0.26						0.07						< 0.01				< 0.01
Usual number of alcohol units consumed																						
No alcohol use	24	11	< 0.01	43	11	< 0.01	151	98	< 0.01	185	100°	< 0.01	134	47	< 0.01	193	85	< 0.01	39	8	53	9
1–2	79	41		234	66		1	1		0			50	18		20	9		166	31	328	46
3–6	65	40		62	22		1	1		0			79	30		15	6		190	37	238	37
> 7	17	9		2	0		0			0			14	5		1	0		97	23	47	8
p**						< 0.01						0.28						< 0.01				< 0.01
Frequency of binge drinking																						
No alcohol use	23	11	< 0.01	42	11	< 0.01	151	98	< 0.01	185	100°	< 0.01	134	47	< 0.01	193	84	< 0.01	39	8	53	9
Never	45	25		215	59		0			0			74	27		23	9		29	6	143	20
Occasionally	98	56		79	28		2	2		0			62	24		14	6		322	65	430	65
Frequently	20	9		4	2		0			0			7	3		0	0		102	22	43	7
P**						< 0.01						0.11						< 0.01				< 0.01
Risky drinking																						
Yes	41	23	< 0.01	58	21	< 0.01	0	0°	< 0.01	0	0°	< 0.01	26	10	< 0.01	8	3	< 0.01	216	45	326	50
p**						0.53												< 0.01				0.13
Lifetime regular smoking																						
Yes	107	55	0.03	105	33	< 0.01	18	8	< 0.01	1	0	< 0.01	106	39	< 0.01	17	7	< 0.01	203	64	209	52
p**						< 0.01						< 0.01						< 0.01				0.01
Current smoking																						
No smoking	170	65	0.04	374	84	0.02	179	89	< 0.01	259	99	< 0.01	224	68	< 0.01	266	93	< 0.01	345	68	517	76
Occasional	15	7		23	7		4	1		1	0		7	2		5	2		56	12	54	9
Daily	66	28		40	10		27	10		2	1		94	29		16	5		83	20	96	15
p**						< 0.01						< 0.01						< 0.01				0.01
Daily consumption of cigarettes																						
None	83	49		238	70		135	92		182	100		182	65		215	94					
1 to 10	41	19		76	25		12	6		1	0		35	13		11	5					
≥ 11	57	32		17	5		5	2		0	0		58	22		3	1					
p**						< 0.01						0.01						< 0.01				
Lifetime use of cannabis																						
Yes	45	21		38	14		0	0°		0	0°		15	6		2	1					
p**						< 0.01												< 0.01				
Cannabis use during the previous 12 months																						
Yes	13	9		4	2		0	0°		0	0°		8	3		0	0°					
p**						< 0.01												< 0.01				
Lifetime use of intravenous drugs																						
Yes	1	8 × 10^−10^		1	9 × 10^−10^		0	0°		0	0°		1	9 × 10^−10^		0	0					
p**						0.53												0.55				

^1^comparison between migrant group and general population by gender

* in comparison to the general population ** between sexes °without age-adjustment

Occasional or frequent binge drinking was reported by 65% of Russian men, 30% of Russian women, 27% of Kurdish men and 6% Kurdish women. These prevalences were significantly smaller than in the general population (87% for men and 72% for women). In general, binge drinking was more frequent in men than in women. The prevalence of risky drinking was lower in all the studied migrant populations relative to the general population (Table 2). Risky drinking was less common among Kurdish women than Kurdish men, but no difference between men and women became evident in Russian or general populations.

### The prevalence of cigarette smoking

The prevalence of lifetime regular smoking was lower in migrant populations than in the general population. The prevalence of lifetime regular smoking and current daily smoking were higher in men than in women in all the studied populations (Table 2). The prevalence of daily smoking was higher in Russian (28%) and in Kurdish (29%) but lower in Somali (10%) men than in the comparison group (20%). In contrast, the prevalence of daily smoking was lower in Russian (10%), Somali (1%) and Kurdish (5%) women compared with the general population (15%). Smoking 11 or more cigarettes per day was reported by 32% of Russian and 22% of Kurdish men. Somali women were excluded from the further analyses because of the low prevalence of smoking.

### The prevalence of illicit drug use

The prevalence of lifetime cannabis use was 21% in men and 14% in women of Russian origin, and lower among Kurdish origin participants (6% in men, 1% in women). Somali participants did not report cannabis use. Somali migrants and Kurdish women were excluded from the further analyses. Cannabis use during the previous 12 months was reported by 9% of Russian men and 2% of Russian women. The lifetime intravenous drug use was very rarely reported in all the migrant groups (Table 2).

### Factors associated with substance use

Socio-demographic and migration-related factors associated with binge drinking, daily smoking and lifetime cannabis use are presented in Table 3 (binge drinking), Table 4 (daily smoking) and Table 5 (lifetime cannabis use), with odds ratios and confidence intervals.

**Table 3:** Factors associated with binge drinking by ethnicity and gender adjusting for age^2^

	Russian		Kurdish		General population	
	Men	Women	Men	Women	Men	Women
	OR (95% CI)	OR (95% CI)	OR (95% CI)	OR (95% CI)	OR (95% CI)	OR (95% CI)
Age						
18–29	2.1 (0.88–4.91)	**4.8 (2.34–9.68)**	**3.0 (1.53–5.87)**	3.1 (0.87–11.09)	1.5 (0.70–3.14)	**1.8 (1.16–2.81)**
30–45	1.3 (0.60–2.91)	**2.4 (1.23–4.83)**	1.6 (0.80–3.09)	1.8 (0.52–6.13)	1.6 (0.84–3.04)	**1.8 (1.20–2.72)**
Over 45	1.0	1.0	1.0	1.0	1.0	1.0
Marital status						
Married or cohabiting	1.0	1.0	1.0	1.0	1.0	1.0
Other	1.3 (0.57–2.79)	1.0 (0.56–1.77)	1.5 (0.88–2.64)	**4.4 (1.92–10.26)**	0.6 (0.32–1.30)	0.9 (0.64–1.33)
Level of basic education						
High school	1.0	1.0	1.0	1.0	1.0	1.0
Secondary school or less	1.4 (0.62–3.29)	1.6 (0.81–3.32)	1.2 (0.72–1.85)	**0.3 (0.14–0.88)**	0.9 (0.49–1.69)	0.8 (0.52–1.19)
Employment situation						
Employed	1.0	1.0	1.0	1.0	1.0	1.0
Unemployed	1.0 (0.45–2.35)	0.8 (0.39–1.72)	**0.5 (0.3–0.9)**	**0.1 (0.02–0.52)**	2.5 (0.56–10.85)	0.9 (0.33–2.27)
Economically inactive	2.8 (0.92–8.43)	0.5 (0.23–1.02)	0.8 (0.48–1.46)	**0.3 (0.13–0.77)**	0.6 (0.27–1.17)	**0.6 (0.42–0.99)**
Economic situation						
Satisfactory	1.0	1.0	1.0	1.0		
Unsatisfactory	**2.5 (1.27–5.01)**	0.7 (0.39–1.20)	0.8 (0.49–1.27)	0.5 (0.19–1.19)		
Age at migration						
Adult	1.0	1.0	1.0	1.0		
Underage	2.4 (0.55–10.43)	2.4 (0.99–5.63)	1.2 (0.63–2.29)	2.1 (0.54–7.92)		
Duration of residence in Finland						
≤ 5 years	1.0	1.0	1.0	1.0		
> 5 years	1.4 (0.61–3.11)	**2.2 (1.08–4.63)**	0.96 (0.55–1.68)	2.6 (0.48–13.69)		
Language proficiency						
Good	1.0	1.0	1.0	1.0		
Fair or less	**0.3 (0.16–0.68)**	0.6 (0.34–1.12)	1.2 (0.78–1.95)	**0.05 (0.01–0.23)**		

^2^Statistically significant findings in bold

**Table 4:** Factors associated with daily smoking by ethnicity and gender adjusting for age^2^

	Russian		Somali	Kurdish		General population	
	Men	Women	Men	Men	Women	Men	Women
	OR (95% CI)	OR (95% CI)	OR (95% CI)	OR (95% CI)	OR (95% CI)	OR (95% CI)	OR (95% CI)
Age							
18–29	**0.4 (0.18–0.83)**	0.9 (0.36–2.34)	1.9 (0.63–5.74)	**1.9 (1.05–3.41)**	3.8 (0.72–19.67)	1.1 (0.59–2.17)	0.8 (0.46–1.47)
30–45	0.7 (0.32–1.34)	1.3 (0.61–2.84)	0.9 (0.30–2.74)	1.2 (0.65–2.12)	**5.0 (1.04–23.96)**	1.1 (0.61–2.02)	1.3 (0.79–2.20)
Over 45	1.0	1.0	1.0	1.0	1.0	1.0	1.0
Marital status							
Married or cohabiting	1.0	1.0	1.0	1.0	1.0	1.0	1.0
Other	**3.6 (1.73–7.60)**	1.5 (0.71–2.96)	**7.0 (2.94–16.56)**	**1.8 (1.08–2.98)**	1.3 (0.53–3.21)	1.5 (0.85–2.80)	**2.2 (1.36–3.58)**
Level of basic education							
High school	1.0	1.0	1.0	1.0	1.0	1.0	1.0
Secondary school or less	**3.3 (1.73–6.36)**	1.6 (0.75–3.42)	**5.1 (1.77–14.63)**	1.2 (0.80–1.84)	0.6 (0.25–1.29)	**3.4 (1.89–6.11)**	**2.5 (1.52–4.01)**
Employment situation							
Employed	1.0	1.0	1.0	1.0	1.0	1.0	1.0
Unemployed	1.5 (0.72–3.25)	1.6 (0.74–3.49)	0.6 (0.23–1.84)	**1.7 (1.06–2.77)**	1.5 (0.59–3.67)	**4.0 (1.58–10.14)**	2.5 (0.90–7.08)
Economically inactive	1.2 (0.51–3.05)	**0.05 (0.01–0.25)**	**0.2 (0.08–0.67)**	0.7 (0.42–1.20)	**0.2 (0.07–0.72)**	0.96 (0.46–2.03)	**0.5 (0.27–0.94)**
Economic situation							
Satisfactory	1.0	1.0	1.0	1.0	1.0		
Unsatisfactory	1.3 (0.67–2.36)	1.3 (0.66–2.55)	**14.8 (4.34–50.63)**	**2.1 (1.30–3.32)**	1.9 (0.66–5.38)		
Age at migration							
Adult	1.0	1.0	1.0	1.0	1.0		
Underage	1.7 (0.54–5.23)	1.0 (0.43–2.43)	1.5 (0.52–4.20)	0.7 (0.39–1.25)	1.5 (0.70–3.03)		
Duration of residence in Finland							
≤ 5 years	1.0	1.0	1.0	1.0	1.0		
> 5 years	1.0 (0.47–2.15)	**3.3 (1.13–9.36)**	**3.6 (1.11–11.58)**	0.9 (0.52–1.40)	1.5 (0.57–4.17)		
Language proficiency							
Good	1.0	1.0	1.0	1.0	1.0		
Fair or less	**2.6 (1.21–5.41)**	0.9 (0.44–1.99)	0.84 (0.30–2.35)	**2.1 (1.32–3.19)**	**0.3 (0.13–0.78)**		

^2^Statistically significant findings in bold

**Table 5:** Factors associated with lifetime cannabis use by ethnicity and gender adjusting for age^2^

	Russian		Kurdish
	Men	Women	Men
	OR (95% CI)	OR (95% CI)	OR (95% CI)
Age			
18–29	**3.9 (1.76–8.71)**	**8.1 (3.72–17.70)**	2.1 (0.88–4.86)
≥30	1.0	1.0	1.0
Marital status			
Married or cohabiting	1.0	1.0	1.0
Other	1.1 (0.44–2.60)	1.0 (0.41–2.26)	2.2 (0.61–7.96)
Level of basic education			
High school	1.0	1.0	1.0
Secondary school or less	1.6 (0.61–4.07)	0.7 (0.23–2.12)	1.9 (0.78–4.52)
Employment situation			
Employed	1.0	1.0	1.0
Unemployed	1.4 (0.48–4.37)	0.7 (0.21–2.42)	2.5 (0.97–6.21)
Economically inactive	2.5 (0.85–7.14)	0.6 (0.24–1.50)	0.6 (0.15–2.43)
Economic situation			
Satisfactory	1.0	1.0	1.0
Unsatisfactory	1.9 (0.81–4.55)	0.8 (0.35–1.70)	1.8 (0.69–4.60)
Age at migration			
Adult	1.0	1.0	1.0
Underage	**7.1 (1.74–28.74)**	1.8 (0.75–4.39)	2.0 (0.71–5.67)
Duration of residence in Finland			
≤ 5 years	1.0	1.0	1.0
> 5 years	1.5 (0.56–3.95)	**2.6 (1.01–6.69)**	3.2 (0.88–11.87)
Language proficiency			
Good	1.0	1.0	1.0
Fair or less	0.7 (0.29–1.73)	0.5 (0.22–1.21)	0.98 (0.44–2.19)

^2^Statistically significant findings in bold

Younger age increased the odds for both binge drinking and daily smoking in Kurdish men and for the lifetime use of cannabis among Russian participants. Not being married or cohabiting increased the odds for binge drinking among Kurdish women, and daily smoking for all migrant men and for women in the general population. Lower basic education decreased the odds for binge drinking for Kurdish women, but increased the odds for daily smoking for Russian and Somali men and both sexes of the general population. Among women, being economically inactive decreased the odds for both binge drinking in Kurdish and general population and daily smoking in Russian, Kurdish and general population. Unemployment decreased the odds for binge drinking in Kurdish men and women, but in contrast increased the odds for daily smoking in men of Kurdish origin and the general population. An unsatisfactory economic situation increased the odds for binge drinking in Russian men, and of daily smoking among Somali and Kurdish men.

Underage migration to Finland in contrast to migration in adulthood increased the odds for lifetime cannabis use in Russian men. A duration of residence in Finland exceeding five years increased the odds for binge drinking, daily smoking and lifetime cannabis use in Russian women, and of daily smoking in Somali men. Poorer language proficiency compared with good language proficiency increased the odds for daily smoking in Russian and Kurdish men, whilst it decreased the odds for daily smoking and binge drinking among Kurdish women, and of binge drinking in Russian men.

## Discussion

This study adds to the limited information on migrants’ substance use in European context. Here, we describe the prevalence and associated background factors on substance use among Russian, Somali and Kurdish migrants in Finland in comparison to the general Finnish population. Our study is the first to examine the habits and background factors of substance use among adult (18–64 years old) Kurdish migrants. The prevalence of alcohol use was lower among migrants compared to the general population, but daily smoking was more prevalent among Russian and Kurdish migrant men than in the comparison group.

### Alcohol consumption

Alcohol use and binge drinking were less frequent, and the proportion of abstainers was larger among all migrant groups compared with the general population. This is in line with previous European studies reporting lower level of alcohol use among migrant populations than among the general populations, especially concerning migrants from non-European countries, and also with previous preliminary findings from the Maamu survey [12–17, 20, 45–48]. The prevalence of alcohol use among Kurdish migrants in our study was lower compared to previous findings on Iranian migrants in Norway [17]. Our results are also in line with previous observations on differences in alcohol use between migrant groups [17, 49]. Based on the data on alcohol use in Russia, the alcohol use of Russian migrants was expected to be higher or similar to the general population [25]. However, the alcohol use among Russian migrants was found to be lower than in the general population. Similar results have been recorded among migrants from the Former Soviet Union in the United States [50]. This could be explained by the higher level of education among Russian migrants compared with the general population, and the selective nature of mainly voluntary immigration from Russia. This may, in part, be explained by the “healthy immigrant effect”, indicating lower levels of substance use among migrant population in comparison to the general population [51, 52]. The prevalence of alcohol use among Kurdish migrants (men 48%, women 15%) has not been previously reported. The prevalence of alcohol use in Somali migrants was low as expected based on cultural and religious factors.

Taken into account the cross-sectional nature of the study, we found that low acculturation was associated with lower levels of binge drinking. Low socioeconomic situation seemed to be both risk and protective factor depending on the migrant group under study, gender and on the individual factors. Lower levels of binge drinking were associated with poor language proficiency among Russian men, shorter duration of residence in Finland among Russian women, unemployment among Kurdish men and a lower basic education and unemployment among Kurdish women. Higher levels of binge drinking were associated with an unsatisfactory economic situation among Russian men, a younger age in Russian women and Kurdish men and marital status being other than married or cohabiting among Kurdish women. Previous literature has reported similar findings between the higher level of acculturation and more frequent binge drinking among men of some migrant groups [53, 54].

### Cigarette smoking

Our findings regarding the larger proportion of daily smokers among Russian and Kurdish migrant men compared to the general population are in concordance with previous European studies and also with preliminary findings from the Maamu survey, reporting a larger proportion of daily smokers among migrants in comparison to the general population [12, 18, 20, 21, 55–57], and in concordance with previous studies reporting differences between migrant groups under study [11, 12, 18, 46, 47, 49, 55, 56, 58–60]. In contrast, previous migrant studies from Spain, Denmark and United States, have reported less smoking or similar smoking behaviour than the general population [15, 16, 50, 60]. It is important to notice, however, that the smoking rates of Finnish men are lower compared to European estimates [33, 34], and the migrant populations under study and definitions of migration differed from one study to another. Compared with the general Finnish population, daily smoking was more common among Russian and Kurdish migrant men, but less prevalent among Kurdish and Somali migrant women, in concordance with previous literature on gender differences in smoking among migrants [56, 60]. Here, the “healthy migrant effect” might apply to women, but not to Russian or Kurdish migrant men. Additionally, differences between lifetime regular smoking and current daily smoking in the general population and Russian migrants might indicate that previous regular smokers have been able to quit smoking, in contrast to Kurdish and Somali migrants among whom this type of pattern is not observed. Similar pattern of lesser smoking cessation among some migrant groups has been reported before, and it might be that there is a gap in educational and smoking cessation services for migrants compared with the services available for the general population [59].

In concordance with previous results, daily smoking among migrant men and men of the general population was associated with poorer socio-economic situation [59, 61]. Daily smoking was associated with not being married or cohabiting in all migrant men. A lower basic education was associated with daily smoking in Russian, and general population men, and unemployment among Kurdish and general population men. Our results on the association of daily smoking and educational level were in line to previous findings from Iraq [32]. An unsatisfactory economic situation was associated with daily smoking among Somali and Kurdish men. Our results imply that a disadvantaged socio-economic position might predispose especially Kurdish and Somali migrant men to daily smoking, taking into account the limitations of cross-sectional studies.

### Lifetime use of cannabis

The prevalence of lifetime cannabis use was low among migrants of Kurdish and Somali origin. The reported prevalence rates of lifetime cannabis use among Russian migrants were smaller compared with those of the general population reported elsewhere [62]. Our results are in line with previous research reporting lower prevalence of cannabis use among migrant adolescents compared with the general population [12, 63]. Lifetime cannabis use was associated with younger age, underage migration to Finland, and a longer residence in Finland in Russian migrants; the implication here being that the adoption of the Finnish culture of substance use could increase the cannabis use among Russian migrants. This is in concordance with previous research reporting that higher level of acculturation might be associated with higher likelihood of cannabis use among migrant adolescents and young adults [19].

### Gender differences

Our results underline the gendered nature of binge drinking and daily smoking in all populations under study, as reported in previous literature [17, 56, 60]. The pronounced gender differences among migrants compared with the general population might reflect the cultural differences in social approval of substance use among women [60, 64]. Economic inactivity was a protective factor for daily smoking and binge drinking among all women, possibly reflecting stay-at-home parenthood. Among Kurdish men, being unemployed seemed to be a protective factor for binge drinking and a risk factor for daily smoking. Russian men showed a trend towards economic inactivity being a risk factor for binge drinking although this finding did not reach statistical significance. Thus, clear gender differences were found in the associations between employment situation and substance use. Being married and having a lower basic education could be interpreted as producing a higher adherence to traditional cultural norms among migrant women. These factors together with poorer language proficiency as an indicator for lower acculturation were all protective factors against binge drinking among Kurdish women. The gender differences in the drinking patterns of Kurdish migrants were reminiscent of the gender differences in drinking patterns in the Finnish population some decades ago; nowadays, this has dissolved with the alarming increase in the alcohol consumption of Finnish women and the simultaneous significant gender role changes in Finnish society [65, 66]. The prevalence of abstainers has decreased and the proportion of weekly drinkers has increased among Finnish women between 1968 and 2008 [67]. Similarly to previous research, we found lower acculturation to be a protective factor against daily smoking in Kurdish women, while it had a contrary effect among Kurdish men [22]. Higher education and adoption of more flexible gender roles as a result of acculturation have been hypothesised to change the patterns of substance use among migrant women, and our results show that this might apply to migrant women of Russian and Kurdish origin [22].

### Strengths and limitations of the study

Our study provides unique data from three different migrant populations to be compared with the general population in one European country (Finland), and it has a relatively high response rate. However, the data used does not allow for analysing the effects of living in rural vs. urban environments. Examining substance use by ethnicity or nationality, as conducted in our study, allows for taking cultural and other variation within the studied populations into account. Reporting substance use among all migrants as one population group or grouping migrants from the same continent as one migrant group may produce observation bias [68]. The comparison between migrant studies may be challenging because different methods used: varying study populations, definitions of migration and migrant groups, and differences in measuring substance use (varying use of standardised questionnaires and definitions) [8]. One of the limitations was that, information was available on frequency of smoking, and no timeframe for “current smoking” was defined. However, self-reported smoking behaviour has been shown to be a reliable measure to use in epidemiological studies [69, 70]. Our study adds to the limited body of literature on habits of substance use among migrant populations, including background factors associated with it. In addition, it is among the first to report the prevalence of substance use among Kurdish migrants. Despite the relatively high response rate and the use of inverse probability weights, the effects of non-response on our results are difficult to estimate. Considerable social stigma is associated with substance use, especially among the Somali participants, and this might have resulted in the under-reporting of substance use among the participants. Various background factors analysed in this study are inter-related, and thus the association of individual factors is difficult to estimate.

## Conclusions

Our results highlight the varying patterns of substance use and differences in associated sociodemographic and migration-related factors between the migrant groups and between the genders within migrant groups. Our results draw attention to the lesser binge drinking among migrants compared with the general population in all the three migrant groups in our study, and to more prevalent daily smoking among Russian and Kurdish migrant men compared to the general population. Therefore in the future, the possible treatment gap for harmful substance use among migrants should be evaluated, and the availability of culturally tailored interventions for smoking cessation [71] and for alcohol use reduction for migrants should be assessed, as proposed in previous literature [8].
